# Relationship between measurements of ipsilateral capitellum and prosthetic radial head size

**DOI:** 10.1186/s13018-022-03393-x

**Published:** 2022-11-19

**Authors:** Weitong Sun, Xieyuan Jiang, Yejun Zha, Maoqi Gong, Ting Li, Kehan Hua, Dan Xiao, Shuai Lu

**Affiliations:** Department of Traumatology and Orthopedics, Beijing Jishuitan Hospital, 31 Xinjiekou East Rd, Xicheng District, Beijing, 100035 China

**Keywords:** Radial head arthroplasty, Capitellum, Three-dimensional reconstruction, Preoperative planning

## Abstract

**Background:**

Selecting the correct size of head component is challenging in radial head arthroplasty, particularly in comminuted fractures. This study aimed to investigate the relationship between measurements of the ipsilateral capitellum and the prosthetic radial head size, which may be used to predict the size of the radial head prosthesis preoperatively.

**Methods:**

Our study enrolled all patients who underwent radial head arthroplasty at Beijing Jishuitan Hospital. Demographic, injury-related and radiographic data were collected. The prosthetic radial head size was recorded from the surgical notes. Three-dimensional models of preoperative CT scans were reconstructed, on which the lateral capitellar diameter, the capitellar width and the width between the capitellum and trochlea were measured. The correlations between measurements of the ipsilateral capitellum and the prosthetic radial head size were evaluated, and linear regression equations were established.

**Results:**

The study enrolled 37 patients, with an average age of 42.8 ± 11.5 years and a male–female ratio of 20:17. The median diameter of the radial head prostheses was 22 (20, 22) mm. The average lateral capitellar diameter was 20.71 ± 1.93 mm, the mean capitellar width was 14.90 ± 1.40 mm, and the mean width between the capitellum and trochlea was 19.29 ± 1.78 mm. The lateral capitellar diameter (*R* = 0.820, *P* < 0.001), the capitellar width (*R* = 0.726, *P* < 0.001) and the width between the capitellum and trochlea (*R* = 0.626, *P* < 0.001) were significantly positively correlated with the size of the radial head prosthesis. The linear regression equation between the lateral capitellar diameter and the size of the radial head prosthesis was calculated and defined as follows: *D* = 7.44 + 0.67*d (*D*: diameter of radial head prosthesis; *d*: lateral capitellar diameter; and adjusted *R*^2^ = 0.719, *P* < 0.001).

**Conclusions:**

There are positive correlations between the anatomical parameters of the ipsilateral capitellum and the prosthetic radial head size. The lateral capitellar diameter can be measured on three-dimensional CT preoperatively to predict the size of the radial head prosthesis intraoperatively.

## Background

Radial head fractures are the most common elbow fractures, accounting for approximately one-third of all elbow fractures [1, 2]. Treatment options include nonsurgical treatment, open reduction and internal fixation, radial head resection and radial head arthroplasty. In recent years, orthopedic surgeons have gradually recognized the important role of the radial head in elbow joint stability [3]. As a result, radial head resection is no longer a routine option for the treatment of severely comminuted radial head fractures [4, 5]. Radial head arthroplasty is gradually becoming the mainstream treatment for this type of injury [6].

The prosthetic size and height are two important factors in determining the clinical outcome of radial head arthroplasty. Biomechanical studies [7–9] have confirmed that an appropriately sized radial head prosthesis can restore the normal mechanical structure of the elbow joint. An undersized radial head prosthesis can lead to postoperative instability, which increases posterior translation with valgus–supination stress [10]. An oversized radial head prosthesis increases radiocapitellar joint stress and alters the biomechanical structure of the proximal radioulnar joint, causing capitellar wear, elbow stiffness and early posttraumatic arthritis [11, 12]. Although previous studies [13–16] have proposed a large number of methods for determining the appropriate height of radial head prostheses, few studies have focused on preoperative planning of the prosthetic size. In clinical practice, orthopedic surgeons generally determine the prosthetic radial head size by measuring the diameter of the contralateral radial head on X-ray or measuring the resected radial head intraoperatively. However, the first approach increases unnecessary X-ray exposure and medical costs, and the second method is less feasible in severely comminuted radial head fractures. Therefore, it is essential to propose a preoperative planning method for predicting the prosthetic radial head size.

Anatomical studies [17] have shown that the radial head size is significantly correlated with the height and width of the capitellum, which makes it possible to predict the size of the radial head prosthesis by preoperatively measuring the anatomical parameters of the ipsilateral capitellum. In recent years, clinical studies [18–20] have proposed that anatomical parameters, such as the humeral condyle diameter, the capitellar width and the capitellum–trochlea width, can be used as predictors of the prosthetic radial head size. However, the optimal predictors and measurement methods remain undetermined. In our study, we measured the anatomical parameters of the ipsilateral capitellum on preoperative three-dimensional computerized tomography (3D-CT) reconstructions with the aim of determining whether there was a correlation between the prosthetic radial head size and anatomical parameters of the ipsilateral capitellum and establishing a preoperative planning method for predicting the prosthetic size.

## Materials and methods

### Subjects

After the institutional research ethics committee approved our retrospective study, we continuously enrolled all patients who underwent radial head arthroplasty at our hospital from January to December 2016. Patients over 14 years old who underwent radial head arthroplasty at our hospital were included. We excluded patients (1) with a congenital elbow deformity, (2) with malunion of a previous elbow fracture, (3) with an ipsilateral capitellar fracture and (4) those whose preoperative computerized tomography (CT) data were not accessible.

A comprehensive search of the medical record database of our hospital was performed with the search term “radial head arthroplasty” between January and December 2016. Demographic data (sex and age), injury-related information (affected side and cause of injury) and surgical notes were collected. In cases of treatment with anatomical prostheses, the long diameter was recorded as the prosthetic diameter. Preoperative CT data were downloaded from the Picture Archiving and Communication Systems (PACS) using the registration number obtained from the search and saved in Digital Imaging and Communications in Medicine (DICOM) format.

### Measurements

The preoperative CT data in DICOM format were loaded using version 17.0 of an interactive medical image control system from Materialise (Materialise, Belgium). We used the CT bone segmentation tool to calculate humeral masks and reconstructed the masks into three-dimensional models.

**Fig. 1:**
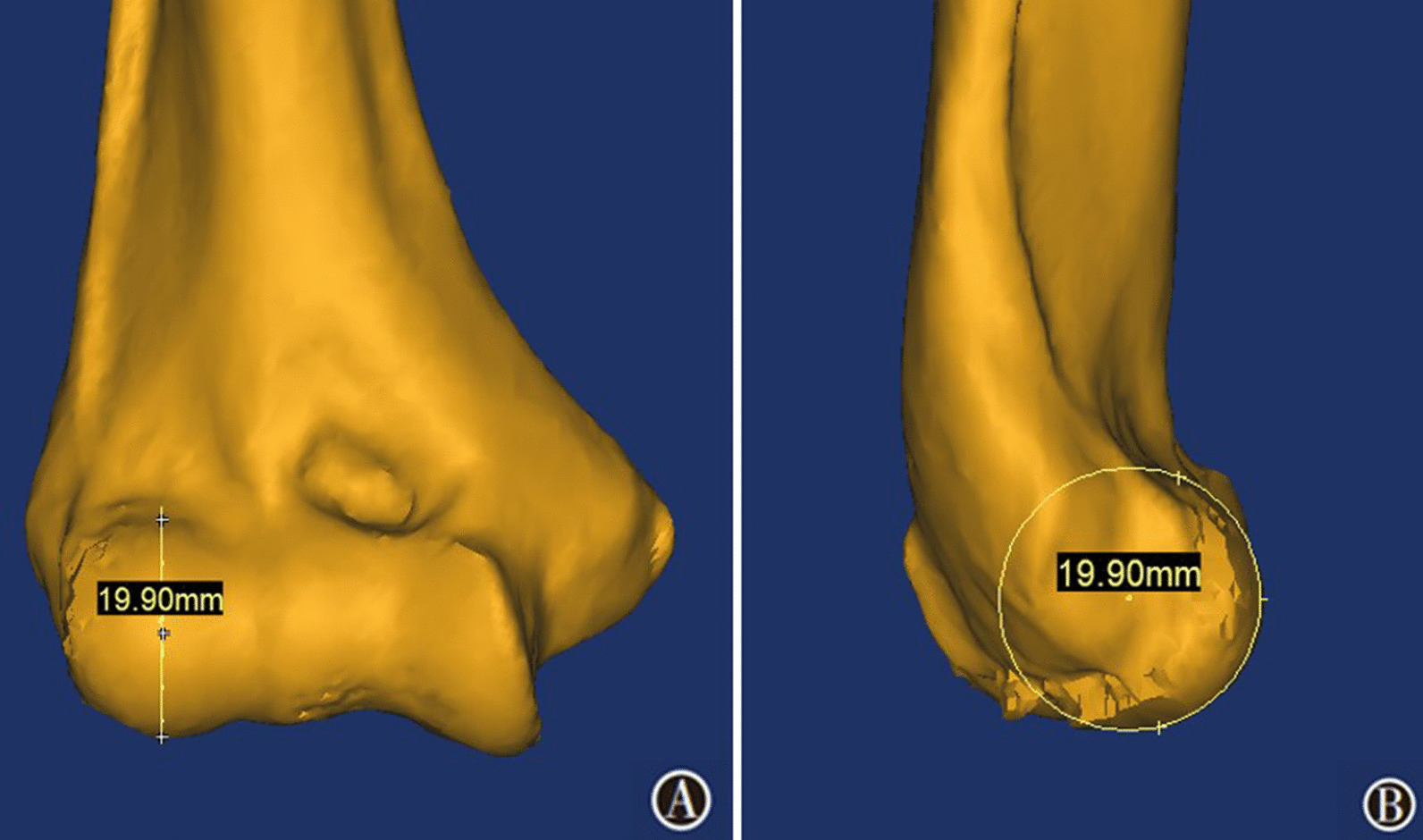
Measurement of the lateral capitellar diameter: **A** Measuring the diameter of the circle identified by the vertex of the superior capitellar edge, the center point of the capitellum and the intersection of the line defined by the above two points with the inferior edge of the capitellum; **B** verifying whether the measured circle was the approximate circle of the capitellar maximum cross section in the lateral view

**Fig. 2:**
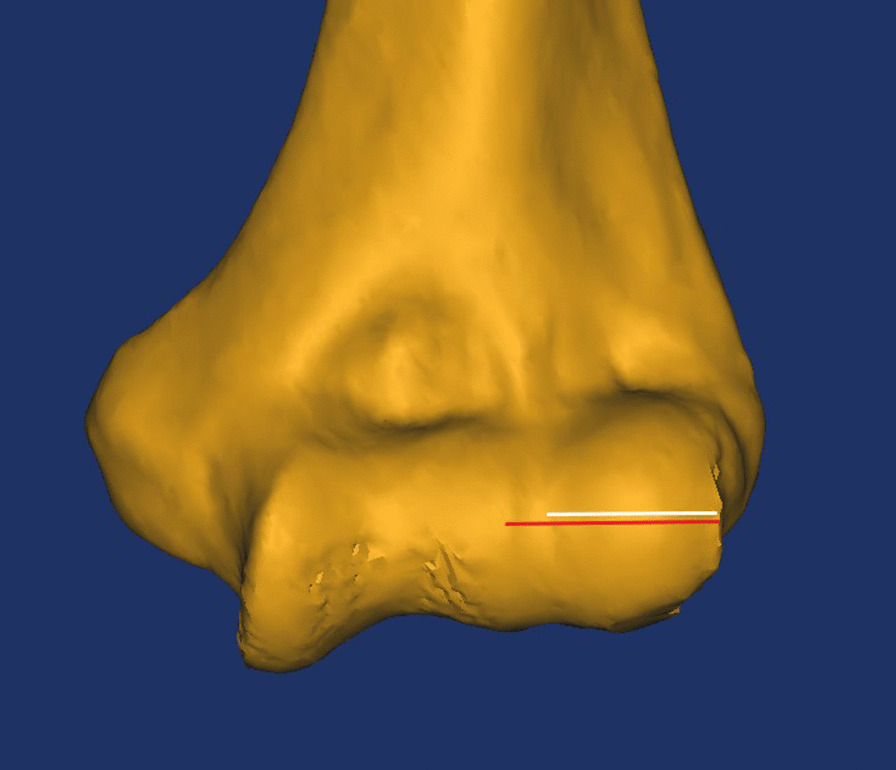
Measuring the capitellar width and the width between capitellum and trochlea: The length of the white line was the capitellar width, and the length of the red line was the width between capitellum and trochlea

On the three-dimensional view, the following capitellar anatomical parameters were measured: the lateral capitellar diameter, the capitellar width and the width between the capitellum and trochlea. While measuring the three anatomical parameters, the position of the distal humeral model should be adjusted on the three-dimensional view to obtain a standard distal humeral anterior–posterior view according to the following criteria: (1) The lateral condyle and medial condyle were fully exposed; and (2) the reconstructed humeral model was adjusted to display the longest axial length. On the standard anterior–posterior view of the distal humeral model, a circle was created using the following three points: the vertex of the superior capitellar edge, the center point of the capitellum and the intersection of the line defined by the above two points with the inferior edge of the capitellum. We defined the diameter of the circle as the lateral capitellar diameter (Fig. 1A), which was measured by the diameter measurement tool in Mimics. This anatomical parameter was the diameter of the approximate circle of the maximum capitellar cross section on the lateral view of the elbow and could be verified by rotating the reconstructed model to the lateral view (Fig. 1B). The capitellar width and the width between the capitellum and trochlea were measured at the equator of the capitellum on the standard anterior–posterior view. The capitellar width was defined as the distance from the lateral edge of the capitellum to the deepest point of the capitello-trochlear groove (the white line in Fig. 2), and the width between the capitellum and trochlea was defined as the distance from the lateral edge of the capitellum to the lateral ridge of the trochlea (the red line in Fig. 2). All parameters were measured independently by two trained orthopedic surgeons, and the average value of each parameter was used for statistical analysis.

### Statistical analysis

Statistical analysis was performed using SPSS version 22.0 statistical software (IBM, USA). All data were tested for normality using the Shapiro–Wilk test. Data with a normal distribution are expressed as *x* ± *s*, and data without a normal distribution are expressed as M (P25, P75). Interobserver reliability was measured with the intraclass correlation coefficient (ICC). A correlation coefficient greater than 0.80 was considered good. The correlations of variables with a bivariate normal distribution were evaluated using the Pearson correlation coefficient; otherwise, the Spearman rank correlation test was used. Linear regression analysis was performed when the two variables were linearly related, independent of each other, and the residuals were normally distributed with equal variance. When *P* < 0.05, the difference was considered statistically significant.

## Results

In our study, we selected all 50 patients who underwent radial head arthroplasty at our hospital from January to December 2016. Thirty-seven patients were enrolled after 13 patients without preoperative CT data were excluded. The mean age of all enrolled patients was 42.8 ± 11.5 years, with a range of 25–74 years. Twenty patients were male, and 17 patients were female. There were 17 left and 20 right elbows treated. Thirty anatomical prostheses (Acumed, Hillsboro, OR, USA) and 7 circumferential prostheses (Wright Medical Technology, Arlington, TN, USA) were used, with a median diameter of 22 (20, 22) mm. The three anatomical parameters of the ipsilateral capitellum and corresponding ICCs are shown in Table 1, indicating that the three measurement methods were all highly reliable.

**Table 1:** Three anatomical parameters

Anatomical parameter	Mean ± SD, mm	Range, mm	ICC (95% CI)	*P* value
Lateral capitellar diameter	20.71 ± 1.93	17.75–25.53	0.987 (0.975–0.993)	< 0.001
Capitellar width	14.90 ± 1.40	11.85–17.48	0.897 (0.768–0.951)	< 0.001
Width between capitellum and trochlea	19.29 ± 1.78	15.62–22.95	0.928 (0.858–0.964)	< 0.001

After the Spearman correlation coefficient was used to evaluate the correlation between the three anatomical parameters and the prosthetic radial head size, it was found that the lateral capitellar diameter (*R* = 0.820, *P* < 0.001), the capitellar width (*R* = 0.726, *P* < 0.001) and the width between the capitellum and trochlea (*R* = 0.626, *P* < 0.001) were positively correlated with the prosthetic radial head diameter, with the strongest correlation between the lateral capitellar diameter and the prosthetic diameter (Table 2).

**Table 2:** Correlation between anatomical parameters and prosthetic size

Anatomical parameter	Spearman correlation coefficient	*P* value
Lateral capitellar diameter	*R* = 0.820	< 0.001
Capitellar width	*R* = 0.726	< 0.001
Width between capitellum and trochlea	*R* = 0.626	< 0.001

**Fig. 3:**
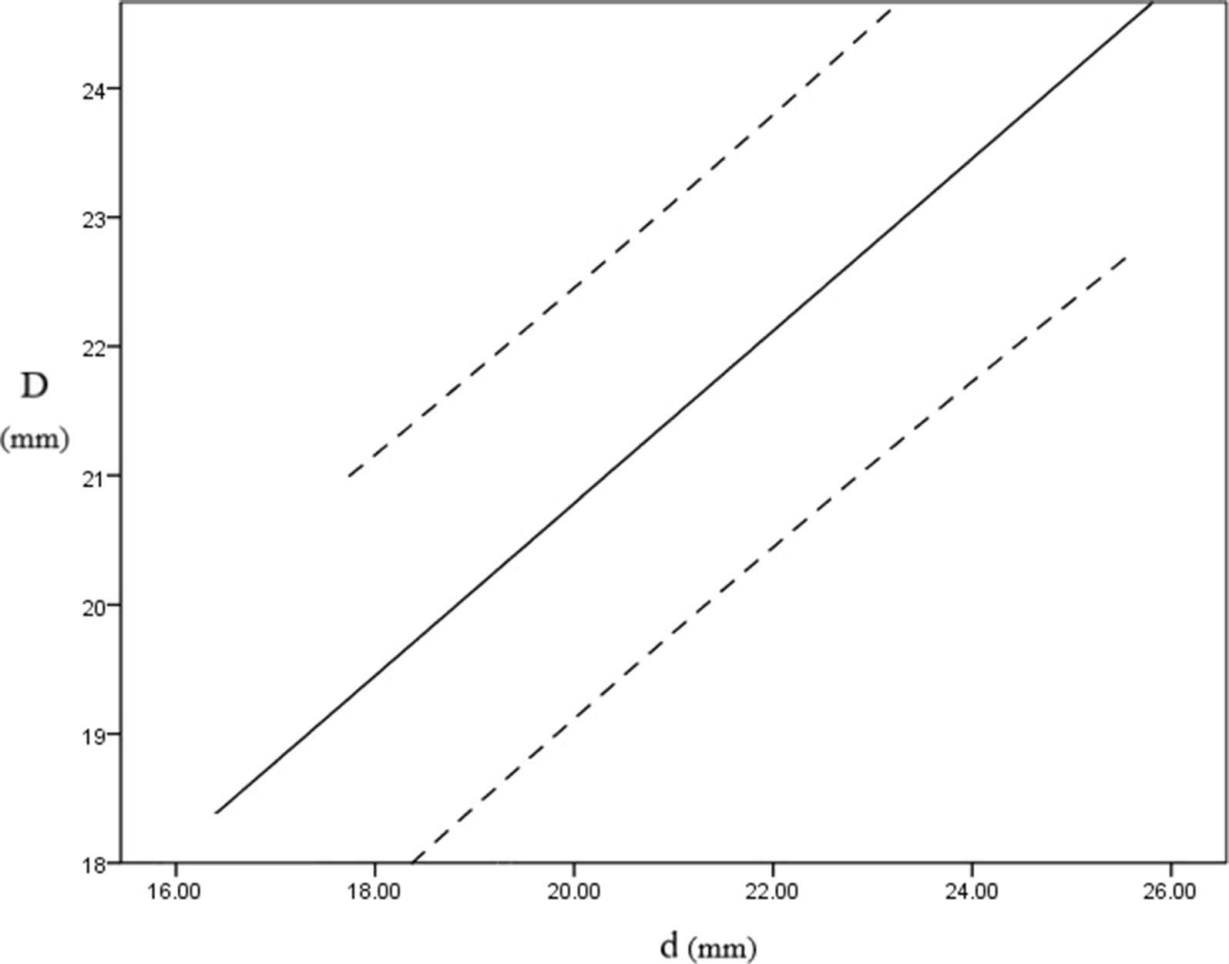
The relationship between prosthetic diameter and lateral capitellar diameter (*D*: prosthetic radial head diameter; *d*: lateral capitellar diameter). The zone between two dashed lines is the 95% confidence interval

Since the lateral capitellar diameter correlated most strongly with the prosthetic radial head diameter, it was possible to predict the size of the radial head implant to be used intraoperatively by measuring the lateral capitellar diameter on the affected side preoperatively. There was a linear relationship between the lateral capitellar diameter and the prosthetic radial head diameter. The following linear regression equation was established: *D* = 7.44 + 0.67*d (*D*: prosthetic radial head diameter; *d*: lateral capitellar diameter; and adjusted *R*^2^ = 0.719, *P* < 0.001, Fig. 3).

## Discussion

The radial head is an important stabilizer resisting elbow valgus stress and forearm axial stress [2, 3], so radial head resection can lead to elbow instability. For instance, some surgeons only focus on radial head fractures when treating forearm injuries that involve the elbow joint, such as Essex-Lopresti injuries, and radial head resection will aggravate the proximal translation of the radius and severely affect the prognosis [21]. For a severely comminuted radial head fracture, internal fixation often compromises postoperative rehabilitation, leading to traumatic elbow stiffness. Therefore, radial head arthroplasty is currently an effective method for the treatment of comminuted radial head fractures, as confirmed by numerous clinical studies [6, 22]. With advances in materials and engineering, types of prostheses have gradually evolved from monoblock to bipolar to modular, and materials have been changed from acrylic resin and silastic to cobalt-chrome, titanium and pyrocarbon [23]. To achieve better upper extremity function after radial head arthroplasty, the focus should be on improving surgical techniques, the main difficulties of which are selecting a prosthesis of an appropriate height and size.

The height of the prosthetic radial head affects the postoperative stability of the elbow. A low prosthetic radial head cannot support the capitellum, resulting in underfilling of the radiocapitellar joint, which causes elbow joint instability. However, a high prosthetic radial head leads to overlengthening of the radius, causing capitellar cartilage erosion, posttraumatic arthritis and elbow stiffness. Numerous studies have attempted to propose intraoperative methods for determining the height of the prosthetic radial head. Doornberg et al. [13] measured the distance between the radial head articular surface and the lateral edge of the coronoid articular surface, suggesting the proximal ulnoradial joint as a reference for optimal insertion of the prosthesis. Athwal et al. [16] performed intraoperative fluoroscopy of the bilateral elbow to determine the prosthetic radial head height, and this method was proven to be practical and reliable. Proper implant size selection is also a determining factor of good outcomes. An oversized prosthesis leads to overfilling of the proximal ulnoradial joint, limiting the range of rotational motion of the forearm. However, there have been few clinical studies on selecting the prosthetic radial head size.

Puchwein et al. [24] measured the diameter of the radial head at different levels in 30 cadaveric elbow joints by 3D-CT and concluded that the commonly used radial head prostheses could meet clinical needs, but that study cannot guide the intraoperative selection of the prosthetic size. Guitton et al. [25] quantified the radial head volume and joint surface area in 50 patients with distal humeral fractures and established multivariate linear regression equations between these two indicators and the radial head diameter, the width of the coronoid process and sex. Although that method established a relationship between the radial head volume and other indicators, it is not useful for predicting the prosthetic radial head size for the following reasons: (1) The prosthetic radial head diameter is more important clinically than the radial head volume; (2) the radial head diameter is an independent variable in the linear regression equation, but in cases of comminuted radial head fractures, the diameter of the affected radial head cannot be measured accurately on preoperative elbow CT; and (3) the anatomical parameters of that method are complicated to measure, cumbersome to calculate, and not suitable for clinical application.

In clinical practice, orthopedic surgeons generally measure the radial head diameter of the contralateral elbow on X-ray preoperatively or the diameter of the resected radial head intraoperatively to determine the size of the selected prosthesis. Rausch et al. [26] reported that the diameters of the contralateral radial head were useful for preoperative estimation of the radial head diameters. However, these methods have the following drawbacks: (1) In patients undergoing radial head arthroplasty, the radial head is usually severely comminuted, and the diameter cannot be measured accurately after resection, and (2) taking contralateral elbow radiographs increases unnecessary X-ray exposure and medical costs. X-ray and CT examination of the affected elbow are necessary in patients undergoing radial head arthroplasty, so it is feasible to use these imaging data to predict the size of the prosthetic radial head. Previous studies have found that anatomical parameters of the ipsilateral capitellum are closely related to the diameter of the radial head. Vanhees et al. [17] measured the anatomical parameters of the radial head and capitellum in 20 cadaveric elbow joints and concluded that the radial head diameter was significantly positively correlated with the vertical height and the anterior width of the capitellum, with Pearson correlation coefficients of 0.8 and 0.9, respectively. Leclerc et al. [18] reconstructed the CT data of 50 normal elbows and found that the capitellar width and the width from the lateral aspect of the capitellum to the lateral trochlear ridge (CAP-TROCHridge) were closely correlated to the maximum and minimum outer diameters of the radial head, with the strongest correlation between CAP-TROCHridge and the maximum outer diameter (*R* = 0.90, *P* < 0.001). Therefore, they recommended CAP-TROCHridge as a predictor of the prosthetic size. Giannicola et al. [19] performed bilateral elbow magnetic resonance imaging on 39 healthy young subjects and found that CAP-TROCHridge and humeral articular width (HUMwidth) were most strongly correlated with the radial head diameters. Vaquero-Picado et al. [20] established a linear regression equation between the prosthetic radial head diameter and the lateral humeral condyle diameter using measurements from the radiographs of 24 patients after radial head arthroplasty. However, that method required a standard preoperative lateral view of the patient’s elbow joint. As elbow injuries often occur with multiple other injuries and patients usually have severe local pain, obtaining satisfactory preoperative radiographs is very difficult. In fact, only 24 of the 32 patients included in that study had X-rays that met the criteria, which limited the clinical application of that method.

Thirty-seven patients were included in our study, which is a sample size similar to that of other studies recently reported in the literature [17–20, 24, 25]. Three-dimensional models of preoperative CT data were reconstructed to measure the anatomical parameters of the capitellum, which allowed adjustment of the distal humeral position after reconstruction and avoided the difficulty of obtaining a standard anterior–posterior/lateral view during radiograph measurement. In our study, three previously reported capitellar anatomical parameters with the strongest correlation with the radial head diameter were selected, namely the lateral capitellar diameter, the capitellar width and the width between the capitellum and trochlea. All three parameters were highly reliable. Statistical analysis showed that the lateral capitellar diameter correlated most strongly with the prosthetic radial head diameter. The lateral capitellar diameter is the diameter of the approximate circle in which the maximum cross section of the capitellum is located on the lateral view of the elbow, which is similar to the capitellar height and the lateral humeral condyle diameter in previous studies [17, 20]. The linear regression equation *D* = 7.44 + 0.67*d (*D*: prosthetic radial head diameter; *d*: lateral capitellar diameter) was established to predict the size of the prosthesis to be used intraoperatively and to improve preoperative planning. This model allows prediction of the prosthetic size from the lateral capitellar diameter in over 70% of cases (72.7%). The relationship between the lateral capitellar diameter and the prosthetic radial head diameter is plotted in Fig. 3 with the 95% confidence interval to assist intraoperative prosthesis sizing.

The limitations of our study include the following two points: (1) Patients treated with anatomical prostheses and circumferential prostheses were both enrolled in this study. The anatomical prosthesis is elliptical, not circular, with slight differences between the long and short diameters, whereas in this study, only the long diameter was used as a parameter of prosthetic size. (2) We only considered anatomical factors, but there are some other factors that influence the prosthetic size, such as preferences of the surgeon and the presence of associated injuries.

## Conclusion

Anatomical parameters of the ipsilateral capitellum are significantly positively correlated with the prosthetic radial head size. The strongest correlation exists between the lateral capitellar diameter and the prosthetic radial head size, with the following linear relationship: *D* = 7.44 + 0.67*d (*D*: prosthetic radial head diameter; *d*: lateral capitellar diameter). Orthopedic surgeons can measure the lateral capitellar diameter on preoperative CT three-dimensional reconstructions to predict the size of the radial head implant used intraoperatively in preoperative planning using the prosthetic radial head size selection graph (Fig. 3).

## Data Availability

The datasets used and/or analyzed during the current study are available from Prof. Xieyuan Jiang on reasonable request.

## Abbreviations

3D-CT: Three-dimensional computerized tomography
CT: Computerized tomography
CAP-TROCHridge: The width from the lateral aspect of the capitellum to the lateral trochlear ridge
DICOM: Digital imaging and communications in medicine
HUMwidth: Humeral articular width
ICC: Intraclass correlation coefficient
PACS: Picture archiving and communication systems
